# Minimal nutrition intervention with high-protein/low-carbohydrate and low-fat, nutrient-dense food supplement improves body composition and exercise benefits in overweight adults: A randomized controlled trial

**DOI:** 10.1186/1743-7075-5-11

**Published:** 2008-04-21

**Authors:** Christopher M Lockwood, Jordan R Moon, Sarah E Tobkin, Ashley A Walter, Abbie E Smith, Vincent J Dalbo, Joel T Cramer, Jeffrey R Stout

**Affiliations:** 1 Metabolic and Body Composition Research Laboratory, Department of Health and Exercise Science, University Of Oklahoma, Norman, OK 73019, USA

## Abstract

**Background:**

Exercise and high-protein/reduced-carbohydrate and -fat diets have each been shown separately, or in combination with an energy-restricted diet to improve body composition and health in sedentary, overweight (BMI > 25) adults. The current study, instead, examined the physiological response to 10 weeks of combined aerobic and resistance exercise (EX) versus exercise + minimal nutrition intervention designed to alter the macronutrient profile, in the absence of energy restriction, using a commercially available high-protein/low-carbohydrate and low-fat, nutrient-dense food supplement (EXFS); versus control (CON).

**Methods:**

Thirty-eight previously sedentary, overweight subjects (female = 19; male = 19) were randomly assigned to either CON (n = 10), EX (n = 14) or EXFS (n = 14). EX and EXFS participated in supervised resistance and endurance training (2× and 3×/wk, respectively); EXFS consumed 1 shake/d (weeks 1 and 2) and 2 shakes/d (weeks 3–10).

**Results:**

EXFS significantly decreased total energy, carbohydrate and fat intake (-14.4%, -27.2% and -26.7%, respectively; *p *< 0.017), and increased protein and fiber intake (+52.1% and +21.2%, respectively; *p *< 0.017). EX and EXFS significantly decreased fat mass (-4.6% and -9.3%, respectively; *p *< 0.017), with a greater (*p *< 0.05) decrease in EXFS than EX and CON. Muscle mass increase only reached significance in EXFS (+2.3%; *p *< 0.017), which was greater (*p *< 0.05) than CON but not EX (+1.1%). Relative VO_2_max improved in both exercise groups (EX = +5.0% and EXFS = +7.9%; *p *< 0.017); however, only EXFS significantly improved absolute VO_2_max (+6.2%; *p *= 0.001). Time-to-exhaustion during treadmill testing increased in EX (+9.8%) but was significantly less (*p *< 0.05) than in EXFS (+21.2%). Total cholesterol and LDL decreased only in the EXFS (-12.0% and -13.3%, respectively; *p *< 0.017). Total cholesterol-to-HDL ratio, however, decreased significantly (*p *< 0.017) in both exercise groups.

**Conclusion:**

Absent energy restriction or other dietary controls, provision of a high-protein/low-carbohydrate and -fat, nutrient-dense food supplement significantly, 1) modified ad libitum macronutrient and energy intake (behavior effect), 2) improved physiological adaptations to exercise (metabolic advantage), and 3) reduced the variability of individual responses for fat mass, muscle mass and time-to-exhaustion – all three variables improving in 100% of EXFS subjects.

## Background

The American College of Sports Medicine (ACSM) Position Stand, "Appropriate intervention strategies for weight loss and prevention of weight regain for adults", states there is little evidence to suggest that exercise-alone is as effective as energy restriction for promoting weight loss [1]. Energy restriction-alone is not, however, a sustainable long-term solution for continued improvements in body composition and health, and instead it has been reported that greater than 50% of subjects that lose weight as a result of dietary restriction-alone eventually regain the weight,[2]. Chronic adjustments to exercise volume and intensity, on the other hand, are virtually limitless. In fact, exercise, in the absence of energy restriction, improves cardiovascular fitness and body composition in a dose-dependent fashion; specifically, decreasing body fat and increasing, or at least preventing significant loss of metabolically active lean body tissue [3].

Another strategy that is gaining support for treating overweight and obesity, either when combined with energy restriction or its absence, is the manipulation of macronutrient composition; more precisely, reducing carbohydrate (CHO) and increasing protein (PRO) intake to improve body composition and blood lipids [4-6]. Krieger et al. [4], for example, concluded that, independent of energy intake, low-carbohydrate/high-protein diets elicit a metabolic advantage; significantly reducing body mass (BM), fat mass, percent body fat and retaining significantly more fat-free mass than diets consisting of greater than 42% of energy from CHO and ≤ 1.05 g/kg/d PRO, respectively. Similarly, Krauss et al. [7], which was further explored by Feinman and Volek [8], showed that in the absence of energy restriction, a reduction in CHO and concomitant increase in PRO and dietary fat (FAT) resulted in significant improvements in BM, total cholesterol, triglycerides, and total cholesterol-to-high-density lipoprotein ratio compared to a diet consisting of 54% CHO, 16% PRO and 30% FAT. Increased satiety and thermogenesis are also commonly reported in response to a high-protein versus normative-protein (~15% of total energy from PRO) diet [9].

Furthermore, it has been reported that the use of 1–2 macronutrient- and micronutrient-containing meal replacements, per day, reduces BM and improves a variety of disease risk factors in overweight and obese populations [10]. Such results, however, have been found to be significantly and positively related solely to the hypocaloric state characteristic of said meal replacement interventions [10]. Nevertheless, a major advantage to the use of a portion-controlled, nutrient-dense food supplement appears to be behavioral in nature – their use minimizes drastic behavior modification and simplifies decision-making [11], resulting in greater long-term compliance [10]. Thus, the purpose to our minimal nutrition intervention, controlled design was to assess if provision of a high-protein/low-carbohydrate and low-fat (~52% PRO, 33% CHO, 15% FAT) nutrient-dense food supplement (Full Strength^®^, Phillips Performance Nutrition, LLC, Golden, CO) would, in the absence of energy restriction, alter *ad libitum *macronutrient intake (behavioral effect) and impose any added metabolic advantage [or detriment] to the physiological effects of 10 weeks of combined aerobic and resistance training in previously sedentary, overweight men and women.

## Methods

### Design, subjects and screening

This study involved a minimal nutritional intervention, controlled design to simulate "real world" use of a high-protein/low-carbohydrate and low-fat, nutrient-dense food supplement while participating in a supervised exercise program for 10 weeks. After screening, participants were randomized into one of three groups: exercise (EX), exercise plus food supplement (EXFS), or control (CON). This study was approved by the University of Oklahoma Institutional Review Board for Human Subjects, and written informed consent was obtained from each participant prior to testing.

Sixty sedentary (< 30 min. physical activity per week), overweight (BMI ≥ 25) men and women volunteered to participate in this study. Six subjects were lost from each of the exercise groups and ten from the CON. Reasons for attrition included lack of time, unwillingness to consume the food supplement (FS) and conflicts with work. Therefore, 38 adult men and women (Table 1) completed the study. Each participant was assessed by routine medical screening for inclusion. None of the participants reported or exhibited: (a) A history of medical or surgical events that may significantly affect the study outcome, including cardiovascular disease, metabolic, renal, hepatic or musculoskeletal disorders; (b) None of the female participants were currently pregnant or breast feeding; (c) Use of any medicine that may significantly affect the study outcome; (d) Use of nutritional supplements, other than a multi-vitamin/mineral, in the four weeks prior to the start of the study; and, (d) Participation in another clinical trial or ingestion of another investigational product within 30 days prior to screening.

**Table 1:** Baseline (PRE) descriptive data of the groups ($\bar{x}$ ± SE).

Variable	**CON** (n = 10; 5F and 5M)	**EX** (n = 14; 7F and 7M)	**EXFS** (n = 14; 7F and 7M)
Age (yr)	30.0 ± 1.6	34.8 ± 1.3	32.6 ± 1.6
Height (cm)	171.7 ± 3.9	175.4 ± 2.3	170.2 ± 2.4
Body Weight (kg)	78.3 ± 5.0	82.3 ± 4.1	84.7 ± 5.5
BMI (kg/m2)	26.5 ± 1.4	26.7 ± 1.2	29.2 ± 1.5

CON = control; EX = exercise only; EXFS = exercise + food supplement. No significant differences were observed between groups for height, weight or BMI (*p *> 0.05).

Prior to the start of the 10-week program (PRE), participants visited the laboratory on two occasions to complete all body composition, cardiorespiratory, strength and blood lipid tests. The same measures were performed during week 12 (POST), after 10 weeks of training. In addition, all participants completed 3-day food logs during PRE and each week of training for a total of 11 weeks. Each food log included two non-consecutive weekdays and one weekend day and was used to represent subjects' average weekly diets. Food logs were analyzed by the same investigator for total energy (kcal), macronutrients and fiber (grams), using Food Processor for Windows, Version 8.6 (ESHA Research, Salem, Oregon). Micronutrient intake was not assessed as part of this investigation.

### Measurements

Height (HT) was measured to the nearest 0.5 cm using a calibrated stadiometer; body mass (BM) was measured using a calibrated clinical scale to nearest 0.01 kg with participants wearing only Spandex shorts or tight-fitting bathing suit. Serum blood samples were drawn at the University of Oklahoma Goddard Health Center. Samples were separated by centrifugation and shipped to Laboratory Corporation of America (Oklahoma City, OK) for analysis. All samples were analyzed using established enzymatic assays for total cholesterol (TC), triglycerides (TRI) and high-density lipoprotein cholesterol (HDL). Low-density and very low-density lipoprotein cholesterol (LDL and VLDL, respectively) were calculated using Friedwald's equations [LDL = TC-TRI/2.2; VLDL = TC-(LDL+HDL)].

### Body composition

All body composition assessments were performed on the same day following a 12-hour fast (*ad libitum *water intake was allowed up to one hour prior to testing). Participants were instructed to avoid exercise for at least 24 hours prior to testing. Fat mass (FM), percent body fat (%FAT) and fat-free mass (FFM) were estimated using the five-compartment (5-C) model described by Wang et al. [12]:

• FM (kg) = 2.748(BV) - 0.715(TBW) + 1.129(Mo) + 1.222(Ms) - 2.051(BM)

• %FAT = (FM/BM) × 100

• FFM = BM - FM

Where BV is total body volume, TBW is total body water, Mo is total body bone mineral, Ms is total body soft tissue mineral, and BM is body mass.

The test-retest reliability for the 5-C equation, as measured 24 to 48 hours apart in 11 men and women, resulted in an intraclass correlation (ICC) of 0.99 and a standard error of measurement (SEM) of 0.48%, 0.36 kg and 0.52 kg for %FAT, FM and FFM, respectively [13]. In addition, there were no significant differences (*p *> 0.05) from trial 1 to trial 2 for %FAT (mean ± SE; 22.0 ± 2.5% to 21.1 ± 2.6%), FM (15.6 ± 1.8 kg to 14.9 ± 1.9 kg) and FFM (55.9 ± 3.4 kg to 56.7 ± 3.5 kg).

Dual-energy X-ray absorptiometry (DXA) (software version 10.50.086, Lunar Prodigy Advance, Madison, WI) was used to estimate total body bone mineral content and total body muscle mass (MM). Bone mineral content (BMC) was converted to Mo using the following equation: Mo = total body BMC × 1.0436 [12]. In addition, the sum of lean soft tissue for both arms and legs (ALST), as measured by DXA, was used to estimate MM from the validated equation of Kim et al. [14]: MM = (1.13 × ALST) - (0.02 × age) + [0.61 × sex (m = 0, f = 1)] + 0.97. Test-retest reliability for MM, as measured 24 to 48 hours apart in 11 male and female subjects resulted in an ICC and SEM of 0.99 and 0.04 kg, respectively [13].

Air displacement plethysmography (BOD POD^®^, Life Measurement, Inc., Concord, CA.) was used to estimate BV. Prior to each test, the BOD POD^® ^was calibrated according to the manufacturer's instructions with the chamber empty and using a cylinder of known volume (49.558 L). The participant, wearing only minimal clothing (as described earlier) and swimming cap, entered and sat in the fiberglass chamber. The BOD POD^® ^was sealed, and the participant breathed normally for 20 seconds while BV was measured. The participant was then connected to a breathing tube internal to the system to measure thoracic gas volume, which was used to correct the BV measurement.

Bioimpedance spectroscopy (BIS) was used to estimate TBW following the procedures recommended by the manufacturer (ImpediMed SFB7, Queensland, Australia). TBW estimates were taken while the participant lay supine on a table with arms ≥ 30 degrees from their torso and legs separated. Electrodes were placed at the distal ends of the participants' right hand and foot. Prior to electrode placement, excess body hair was removed, and the skin at each site was cleaned with alcohol. Prior to analysis, each participant's HT, BW, age and sex were entered into the BIS device. Internal to the device, the BIS utilized 256 frequencies to estimate TBW. The average of two trials within ± 0.05 liters was used to represent each participant's TBW. The TBW estimate was then used to estimate Ms using the equation from Wang et al. [12]: Ms = TBW × 0.0129. The BIS device used in the current study was recently examined in our laboratory as compared to deuterium oxide for estimating TBW in a heterogeneous sample of men and women (*n *= 30; 23.8 ± 4 yrs; 174.47 ± 7.34 cm; 73.4 ± 18.45 kg; 23.10 ± 5.77 %FAT; $\bar{x}$ ± SD). The results demonstrated a non-significant constant error (CE = -0.56L, *p *> 0.05) and high correlation (*r *= 0.97), which is similar to results obtained in other laboratories [15-17]. Therefore, we feel confident that our BIS device accurately estimated TBW.

### Cardiorespiratory measurements

During a graded exercise test (GXT) on a Quinton^® ^Q65 Series 90 Treadmill (Quinton Instrument Co., Seattle, WA), respiratory gases were monitored and continuously analyzed with open-circuit spirometry to calculate minute ventilation (VE), oxygen consumption rate (VO_2_), carbon dioxide expiration rate (VCO_2_) and respiratory exchange ratio (RER) using a metabolic cart and manufacturer's software (True One 2400^®^, Parvo-Medics, Inc., Provo, UT). The data were averaged over 30-second intervals. Prior to each test, the metabolic cart was calibrated using room air for the flow rate calibration and gases of known volume and concentration for the calibration of the O_2 _and CO_2 _analyzers. The highest 30-second VO_2 _value during the GXT was recorded as the maximal oxygen uptake (VO_2_max) if it coincided with at least two of the following criteria: (a) Plateau in heart rate (HR) or HR values within 10% of the age-predicted HR_max_; (b) Plateau in VO_2 _(defined as an increase of not more than 150 ml/min); and/or, (c) RER value greater than 1.15. Test-retest reliability for VO_2_max, as measured using 10 male and female subjects, resulted in an ICC and SEM of 0.98 and 1.17 ml/kg/min, respectively.

### Strength measurements

Each participant completed a 5-repetition maximum (5RM) for upper- and lower-body strength (bench press and squat, respectively), using a Cybex^® ^Plate Loaded Smith Press (Cybex International, Medway, MA). Each participant completed a familiarization session prior to testing. Testing began with a warm-up consisting of 8 to 10 repetitions at approximately 50% of the tester-estimated 5RM load. Following adequate rest of 2 to 3 minutes, weight was added and participants attempted five repetitions through the full range of motion. After each successful set of five repetitions, the weight was increased until subjects could no longer complete five repetitions. Participants rested for 2 to 3 minutes between sets. Test-retest, for bench press and squat, as measured using 10 male and female subjects, resulted in an ICC and SEM of 0.99, and 0.83 kg and 1.4 kg, respectively.

### Training protocol

The exercise program was designed using the American College of Sports Medicine (ACSM) recommended guidelines for apparently healthy adults; all participants were supervised and trained by an ACSM or National Strength and Conditioning Association certified trainer. Endurance training was performed three days per week. Participants were allowed to select the mode of exercise, provided it utilized large muscle groups and was rhythmic in nature. The progressive endurance training program used is summarized in Table 2. Resistance training was performed two days per week, providing at least 24 hours recovery between exercise sessions. Participants completed 10 exercises that incorporated all major muscle groups. Each exercise was performed once per session, and participants completed 8 to 12 repetitions per exercise until volitional exhaustion. Weight was increased when participants performed 12 repetitions at the same resistance during two consecutive lifting sessions. All lifts were performed on Nautilus Nitro^® ^(Nautilus, Inc., Vancouver, WA) selectorized resistance machines.

**Table 2:** Endurance training protocol

Week	Duration (min)	% Heart Rate Reserve
1	15 – 20	40 – 50
2	20 – 25	40 – 50
3	25 – 30	50 – 60
4	25 – 30	50 – 60
5	25 – 30	60 – 70
6	25 – 30	60 – 70
7	25 – 30	60 – 70
8	30 – 35	60 – 70
9	30 – 35	60 – 70
10	30 – 35	60 – 70

### Nutritional protocol

**Figure 1:**
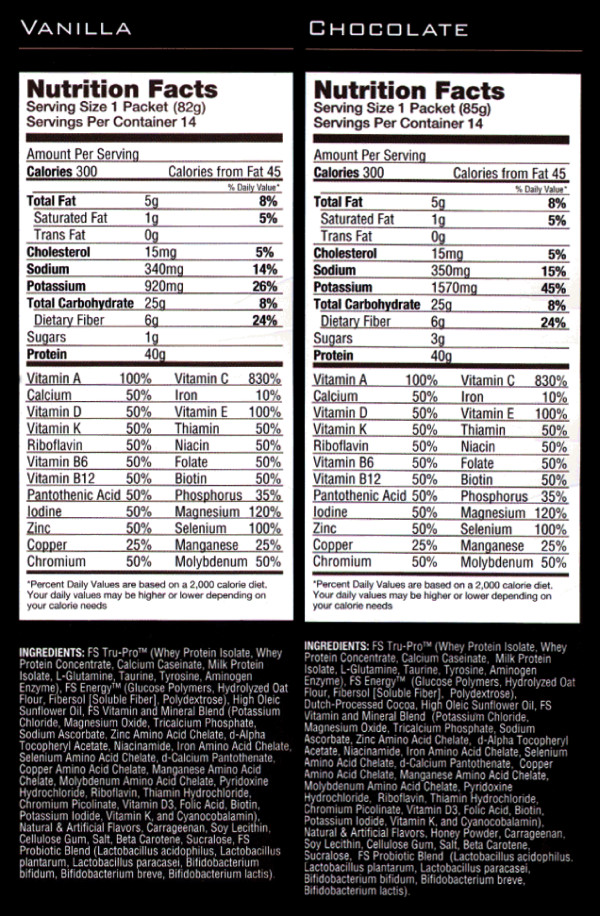
**Nutrition Facts and Ingredients of the Food Supplement (Full Strength^®^, Phillips Performance Nutrition, Golden, CO)**. Subjects in EXFS (exercise + food supplement) consumed 1FS/d (Week 1 and 2) and 2FS/d (Week 3–10) in combination with *ad libitum *diet. Participants were free to choose either chocolate- or vanilla-flavored FS. Two-week supplies of FS were provided at bi-weekly weigh-ins.

Participants in the EXFS group were instructed to consume one FS per day (Full Strength^®^, Phillips Performance Nutrition, LLC, Golden, CO) (Figure 1) for the first two weeks, and two servings per day for the remaining eight weeks. Subjects were provided a two-week supply of FS at the end of testing during PRE and at biweekly weigh-ins throughout the duration of the study. Time of day for consuming the FS, as well as how to incorporate the FS into each subject's diet (i.e. consuming as an additional meal or in place of another meal), was left to the subject's choosing. Participants were instructed to mix the FS in accordance with the label directions. All participants in CON, EX and EXFS were advised to maintain current (*ad libitum*) diet.

### Statistical analyses

Separate two-way mixed factorial ANOVAs [group (CON vs. EX vs. EXFS) × time (PRE vs. POST)] were used to identify any group × time interactions. If a significant interaction was observed, the statistical model was decomposed by examining the simple main effects with one-way repeated measures ANOVAs for each group and one-way factorial ANOVAs for each time. In the event of significant simple main effects, Tukey *post-hoc *comparisons were performed among the groups; all pair-wise comparison dependent samples t-tests with Bonferroni corrections (*p *≤ 0.017) were performed across time. If there was no interaction, main effects were analyzed by collapsing across the non-interacting variable as described above for simple main effects. The level of significance was set at *p *≤ 0.05. Analyses were performed using SPSS 14.0 (SPSS Inc. Chicago, IL). SPSS-derived *p*-values of less than 10^-3 ^(i.e., "*p *= 0.000") are reported in the manuscript as the critical *p*-value necessary to obtain significance (i.e., "*p *≤ 0.05" or "*p *≤ 0.017"). Statistical power calculations demonstrated power in this investigation ranged from 0.69 to 0.92.

## Results

### Nutritional profile

**Figure 2:**
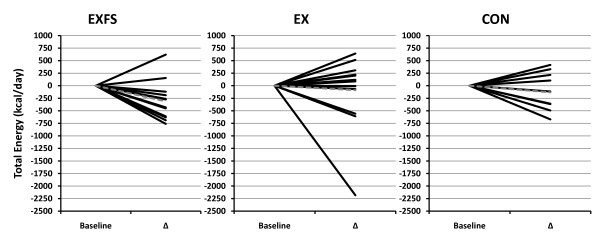
**Change in Average Daily Energy Intake by Subject**. Individual responses for average daily intake of Total Energy from Baseline to Week 3–10 (Δ). Exercise + FS (EXFS; left); Exercise-alone (EX; center); Control (CON; right). Dashed line represents the mean. Total Energy was significantly reduced over time (*p *≤ 0.017) in EXFS.

**Figure 3:**
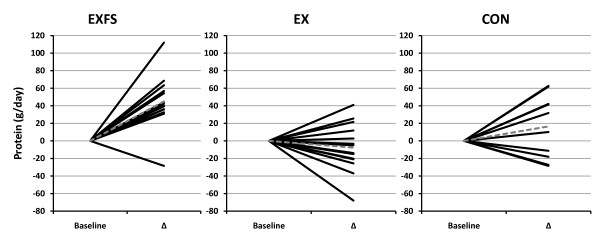
**Change in Average Daily Protein Intake by Subject**. Individual responses for average daily intake of Protein from Baseline to Week 3–10 (Δ). Exercise + FS (EXFS; left); Exercise-alone (EX; center); Control (CON; right). Dashed line represents the mean. Protein intake was significantly increased over time (*p *≤ 0.017) in EXFS, and significantly greater than EX (*p *≤ 0.05).

**Figure 4:**
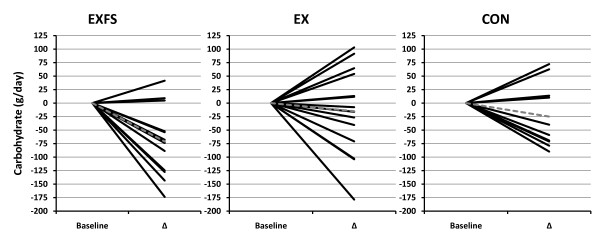
**Change in Average Daily Carbohydrate Intake by Subject**. Individual responses for average daily intake of Carbohydrate from Baseline to Week 3–10 (Δ). Exercise + FS (EXFS; left); Exercise-alone (EX; center); Control (CON; right). Dashed line represents the mean. Carbohydrate intake was significantly reduced over time (*p *≤ 0.017) in EXFS.

Baseline measures, including estimated dietary intake did not differ (*p *> 0.05) among subjects in each of the groups (Table 1 and 3, 4, 5, 6, 7). Estimated mean daily energy intake did not differ (*p *> 0.05) between groups, however, a significant decrease did occur for total energy within EXFS from PRE to Week 3–10 (-14.4%; *p *= 0.002); no significant changes appeared within EX or CON. Individual analysis revealed 85.7% (n = 12) of subjects in EXFS reduced average energy intake by -118.62 to -761.12 kcal/d, with only two subjects reporting increases from PRE to Week 3–10; 21.4% (n = 3) of EX subjects reduced energy intake by -557.87 to -2,184.50 kcal/d (Figure 2). A two-way interaction (*p *= 0.001; 1 - β = 0.97) was identified for mean protein intake from PRE to Week 3–10 (Table 3). *Post-hoc *analysis revealed the change in protein intake for EXFS was significantly greater than EX (+52.1% versus -9.2%, respectively; *p *≤ 0.05). Individual analysis revealed 92.9% (n = 13) of subjects in EXFS increased protein intake by +31 to +112.13 g/d, whereas EX values ranged from -68 to +41 g/d (Figure 3). No difference (*p *> 0.05) was identified for mean total carbohydrate and fiber intake among groups; however, a significant decrease (-27.2%; *p *= 0.002) in mean daily carbohydrate and an increase (+21.2%; *p *≤ 0.017) in mean daily fiber intake did occur within EXFS from PRE to Week 3–10, while no significant changes appeared in EX or CON (Table 3). Individual analysis revealed that 78.6% (n = 11) of subjects in EXFS reduced mean carbohydrate intake by -52.25 to -173.62 g/d (Figure 4). There was no difference (*p *> 0.05) for mean daily fat intake among groups; however, there was a significant decrease in mean daily fat intake within EXFS from PRE to Week 3–10 (-26.7%; *p *≤ 0.017).

**Table 3:** Average daily energy and macronutrient intake from PRE to Week 1–2 and Week 3–10 ($\bar{x}$ ± SE).

	**PRE**	**Week 1–2**	**Week 3–10**
Total Energy Intake (kcal/d)*			
CON	2076 ± 168.0	1853 ± 167	1945 ± 66
EX	2039 ± 196.0	2021 ± 127	1951 ± 115
EXFS	2166 ± 160.0	1865 ± 99^a^	1854 ± 98^a^
Total Protein Intake (g/d)**			
CON	84.1 ± 8.8	90.9 ± 8.8	90.6 ± 11.6
EX	82.4 ± 5.9	75.8 ± 5.2	74.8 ± 5.1
EXFS	86.3 ± 8.5	102.2 ± 3.3	131.3 ± 4.0^a,b,c^
Total Carbohydrate Intake (g/d)*			
CON	253.0 ± 23.1	205.1 ± 19.0	238.0 ± 16.6
EX	268.4 ± 23.1	263.1 ± 19.4	253.5 ± 20.3
EXFS	261.5 ± 20.9	205.0 ± 19.4^a^	190.3 ± 10.5^a^
Total Fat Intake (g/d)*			
CON	80.9 ± 6.7	74.3 ± 7.0	70.1 ± 5.5
EX	70.6 ± 10.6	73.9 ± 5.7	70.7 ± 4.3
EXFS	86.1 ± 7.7	70.7 ± 4.5^a^	63.1 ± 4.3^a,b^
Total Fiber Intake (g/d)**			
CON	15.7 ± 1.3	15.3 ± 1.7	16.8 ± 2.1
EX	16.3 ± 1.8	16.5 ± 1.6	15.5 ± 1.8
EXFS	18.9 ± 2.1	19.5 ± 0.8	22.9 ± 0.9^a,b^

Representative daily intake was calculated as the mean of values as determined from 3-day food logs, recorded weekly by each participant. CON = control; EX = exercise only; EXFS = exercise + food supplement. One shake per day, was consumed during Week 1–2; two shakes, per day, were consumed during Week 3–10. *Main effect for time (*p *≤ 0.05); **Group-by-Time interaction (*p *≤ 0.05). ^a^Different from PRE (*p *≤ 0.017); ^b^Different from Week 1–2 (*p *≤ 0.05); ^c^Different from EX (*p *≤ 0.05).

**Table 4:** Changes in body composition from PRE to POST ($\bar{x}$ ± SE).

	**PRE**	**POST**	**CHANGE**
Body Mass (kg)*			
CON	78.2 ± 5.0	77.9 ± 5.0	-0.3 ± 0.5
EX	82.3 ± 4.1	82.0 ± 4.0	-0.3 ± 0.5
EXFS	84.7 ± 5.5	82.9 ± 5.2	-1.8 ± 1.0
Fat Mass (kg)**			
CON	22.6 ± 2.0	22.2 ± 2.0	-0.4 ± 0.4
EX	23.9 ± 2.2	22.8 ± 2.9	-1.1 ± 0.4^a^
EXFS	28.9 ± 2.3	26.2 ± 2.3	-2.7 ± 0.4^a,b,c^
% Body Fat**			
CON	28.9 ± 2.4	28.5 ± 2.6	-0.4 ± 0.4
EX	29.0 ± 2.2	27.8 ± 2.3	-1.2 ± 0.4^a^
EXFS	34.1 ± 1.3	31.6 ± 1.4	-2.5 ± 0.4^a,b,c^
Fat-Free Mass (kg)*			
CON	55.6 ± 4.3	55.7 ± 4.4	0.10 ± 0.3
EX	58.4 ± 3.5	59.2 ± 3.7	0.80 ± 0.6
EXFS	55.8 ± 3.5	56.7 ± 3.4	0.90 ± 0.5
Muscle Mass (kg)**			
CON	26.8 ± 2.5	26.8 ± 2.6	0.0 ± 0.1
EX	28.1 ± 2.2	28.4 ± 2.3	0.3 ± 0.2
EXFS	26.5 ± 2.1	27.1 ± 2.1	0.6 ± 0.1^a,b^

CON = control; EX = exercise only; EXFS = exercise + food supplement. *Main effect for time (*p *≤ 0.05); **Group-by-Time interaction (*p *≤ 0.05). ^a^Different from PRE (*p *≤ 0.017); ^b^Different from CON (*p *≤ 0.05); ^c^Different from EX (*p *≤ 0.05).

**Table 5:** Changes in upper- and lower-body strength from PRE to POST ($\bar{x}$ ± SE).

	**PRE**	**POST**	**CHANGE**
Bench Press (kg)**			
CON	49.9 ± 8.8	51.3 ± 9.0	1.4 ± 0.4
EX	47.7 ± 7.4	54.0 ± 5.7	6.3 ± 0.8^a,b^
EXFS	50.0 ± 6.4	58.5 ± 7.2	8.5 ± 1.0^a,b^
Squat (kg)**			
CON	60.9 ± 8.4	61.9 ± 8.0	1.0 ± 0.6
EX	59.2 ± 7.2	70.8 ± 7.3	11.6 ± 1.4^a,b^
EXFS	61.0 ± 6.9	74.9 ± 7.2	13.9 ± 1.8^a,b^

Upper- and lower-body strength, as assessed by five-repetition maximum (5RM) bench press (upper) and squat (lower). CON = control; EX = exercise only; EXFS = exercise + food supplement. *Main effect for time (*p *≤ 0.05); **Group-by-Time interaction (*p *≤ 0.05). ^a^Different from PRE (*p *≤ 0.017); ^b^Different from CON (*p *≤ 0.05).

**Table 6:** Changes in cardiorespiratory fitness from PRE to POST ($\bar{x}$ ± SE).

	**PRE**	**POST**	**CHANGE**
VO_2_max (ml·kg^-1^·min^-1^)**			
CON	36.8 ± 2.1	36.9 ± 2.0	0.1 ± 0.5
EX	35.7 ± 2.7	37.5 ± 2.4	1.8 ± 0.6^a^
EXFS	32.9 ± 1.9	35.5 ± 1.8	2.6 ± 0.4^a^
VO_2 _(L·min^-1^)*			
CON	2.87 ± 0.25	2.88 ± 0.25	0.01 ± 0.01
EX	2.87 ± 0.23	3.00 ± 0.82	0.13 ± 0.05
EXFS	2.76 ± 0.20	2.93 ± 0.21	0.17 ± 0.04^a^
Minute Ventilation (L·min^-1^)*			
CON	79.4 ± 7.6	79.0 ± 6.0	0.4 ± 2.5
EX	83.3 ± 5.0	89.1 ± 5.5	5.8 ± 2.4^a^
EXFS	83.7 ± 4.7	88.0 ± 5.1	4.3 ± 1.4^a^
Maximum Heart Rate (bpm)*			
CON	192.1 ± 2.7	190.9 ± 2.0	-1.2 ± 1.8
EX	190.6 ± 2.6	188.6 ± 2.8	-2.0 ± 1.3
EXFS	187.0 ± 6.2	184.6 ± 6.0	-2.4 ± 1.5
Time-to-Exhaustion (sec)**			
CON	643.9 ± 44.8	653.0 ± 46.4	9.1 ± 13.4
EX	681.8 ± 62.0	748.6 ± 65.4	66.8 ± 13.8^a,b^
EXFS	559.9 ± 46.8	678.6 ± 50.1	118.7 ± 14.3^a,b,c^

Cardiorespiratory fitness, as assessed by graded treadmill test to exhaustion. CON = control; EX = exercise only; EXFS = exercise + food supplement. *Main effect for time (*p *≤ 0.05); **Group-by-Time interaction (*p *≤ 0.05). ^a^Different from PRE (*p *≤ 0.017). ^b^Different from CON (*p *≤ 0.05). ^c^Different from EX (*p *≤ 0.05).

**Table 7:** Changes in fasting lipid concentrations from PRE to POST ($\bar{x}$ ± SE).

	**PRE**	**POST**	**CHANGE**
Total Cholesterol (mg/dL)*			
CON	179.8 ± 11.4	173.2 ± 10.9	-6.6 ± 6.6
EX	186.4 ± 7.3	178.2 ± 7.1	-8.2 ± 3.4
EXFS	197.5 ± 10.1	173.9 ± 13.1	-23.6 ± 6.7^a^
HDL (mg/dL)*			
CON	53.9 ± 3.4	51.3 ± 2.4	-2.6 ± 1.8
EX	45.1 ± 2.2	41.9 ± 2.0	-3.2 ± 1.5
EXFS	51.3 ± 4.1	50.9 ± 4.2	-0.7 ± 1.6
LDL (mg/dL)*			
CON	107.3 ± 8.7	99.8 ± 8.7	-7.5 ± 4.5
EX	119.7 ± 7.3	112.9 ± 5.8	-6.8 ± 2.7
EXFS	119.5 ± 8.4	103.6 ± 10.5	-15.9 ± 5.2^a^
TC:HDL*			
CON	3.6 ± 0.3	3.3 ± 0.3	-0.3 ± 0.1
EX	4.6 ± 1.3	4.2 ± 1.2	-0.4 ± 0.1^a^
EXFS	4.2 ± 1.3	3.8 ± 1.2	-0.4 ± 0.1^a^
VLDL (mg/dL)*			
CON	21.2 ± 3.8	20.0 ± 3.1	-1.2 ± 1.8
EX	24.7 ± 3.2	20.9 ± 2.9	-3.8 ± 2.6
EXFS	25.6 ± 3.9	22.3 ± 3.4	-3.3 ± 2.2
Triglycerides (mg/dL)*			
CON	106.7 ± 18.6	98.1 ± 13.9	-8.6 ± 8.5
EX	124.0 ± 16.1	104.1 ± 14.4	-19.9 ± 12.9
EXFS	138.0 ± 19.0	111.2 ± 17.0	-26.8 ± 10.0

CON = control; EX = exercise only; EXFS = exercise + food supplement. HDL = high-density lipoproteins; LDL = low-density lipoproteins; TC:HDL = total cholesterol-to-high-density lipoproteins ratio; VLDL = very low-density lipoproteins. *Main effect for time (*p *≤ 0.05). ^a^Different from PRE (*p *≤ 0.017).

### Body composition

**Figure 5:**
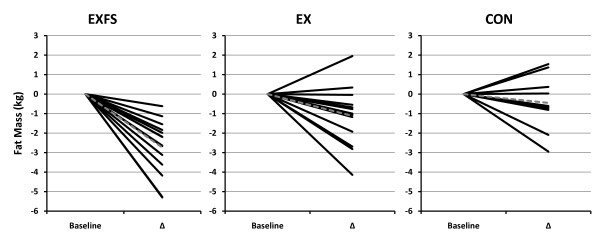
**Change in Fat Mass by Subject**. Individual responses for changes in Fat Mass from Baseline to Post Week 3 (Δ). Exercise + FS (EXFS; left); Exercise-alone (EX; center); Control (CON; right). Dashed line represents the mean. Fat Mass was significantly reduced over time (*p *≤ 0.017) in EXFS, and reduced significantly more than EX (*p *≤ 0.05).

**Figure 6:**
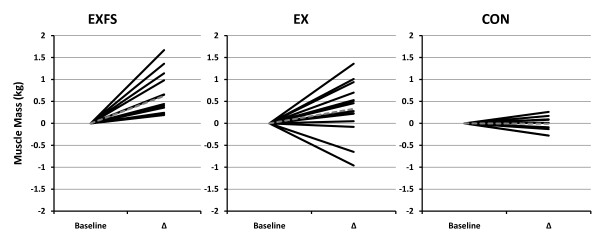
**Change in Muscle Mass by Subject**. Individual responses for changes in Muscle Mass from Baseline to Post Week (Δ). Exercise + FS (EXFS; left); Exercise-alone (EX; center); Control (CON; right). Dashed line represents the mean. Muscle Mass increased significantly over time (*p *≤ 0.017) in EXFS, and was significantly greater than CON (*p *≤ 0.05).

A significant (*p *= 0.044; 1 - β = 0.61) main effect for time was observed across all groups for BM and FFM; however, dependent t-tests showed no significant change for any single group (Table 4). A two-way interaction (*p *= 0.002; 1 - β = 0.94) was identified for %FAT and FM from PRE to POST; *post-hoc *analyses revealed significantly greater decreases in %FAT and FM for EXFS (-2.5% and -2.7 kg, respectively; *p *≤ 0.05) than in EX (%FAT = -1.2%; FM = -1.1 kg) and CON (%FAT = -0.4%; FM = -0.4 kg). In addition, there was a two-way interaction (*p *= 0.014; 1 - β = 0.77) for MM from PRE to POST. The increase in MM was significantly (*p *= 0.05) greater in EXFS (+2.3%) than CON (0.0%) but did not differ significantly from EX (+1.1%); EX was not significantly different (*p *> 0.05) from CON (Table 4). Individual analysis revealed that 100% (n = 14) of subjects in EXFS reduced FM (range = -0.62 to -5.3 kg) and increased MM (range = +0.19 to +1.67 kg); 85.7% (n = 12) of subjects in EX reduced FM (range = -0.04 to -4.14 kg) and 78.6% increased MM (range = +0.05 to +1.36 kg) (Figures 5 and 6).

### Strength measurements

There was a two-way interaction (*p *= 0.001; 1 - β = 0.99) for 5RM strength for both the bench press and squat (Table 5). Upper- and lower-body strength significantly improved for EX (+13.2% and +19.6%; *p *≤ 0.017) and EXFS (+17.0% and +22.8%; *p *≤ 0.017), with no changes (*p *> 0.05) in CON. No difference (*p *> 0.05) was evident between EX and EXFS.

### Cardiorespiratory measurements

**Figure 7:**
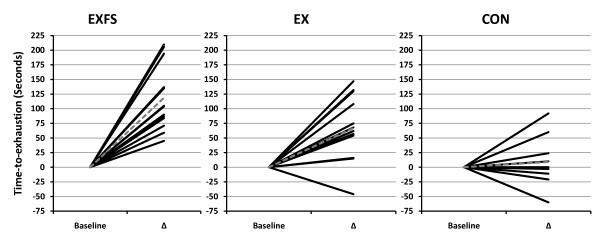
**Change in Time-to-Exhaustion by Subject**. Individual responses for Time-to-Exhaustion (TTE) from Baseline to Post (Δ). Exercise + FS (EXFS; left); Exercise-alone (EX; center); Control (CON; right). Dashed line represents the mean. TTE increased significantly over time (*p *= 0.001) in EXFS; EXFS was significantly greater than EX and CON (*p *≤ 0.05).

VO_2_max (ml/kg/min) improved (*p *≤ 0.017) for EX (+5.0%) and EXFS (+7.9%), however, no changes were observed for CON (Table 6). When expressed in absolute terms (L/min), VO_2_max increased significantly in EXFS (+6.2%; *p *= 0.001), whereas EX demonstrated a non-significant (*p *> 0.017) increase of 4.5%. There was a two-way interaction (*p *= 0.001; 1 - β = 0.99) for time-to-exhaustion (TTE). *Post-hoc *analysis determined that increases in TTE observed in the EX (+9.8%) and EXFS (+21.2%) groups were significantly greater (*p *≤ 0.05) than changes in CON (+1.4%); the improvement in TTE was significantly greater (*p *≤ 0.05) in EXFS than EX. Individual analysis revealed 100% (n = 14) of EXFS improved TTE (+45 to +210 sec), versus 92.9% (n = 13) of EX (-46 to +147 sec) and 40% (n = 4) of CON (Figure 7).

### Blood lipids

**Figure 8:**
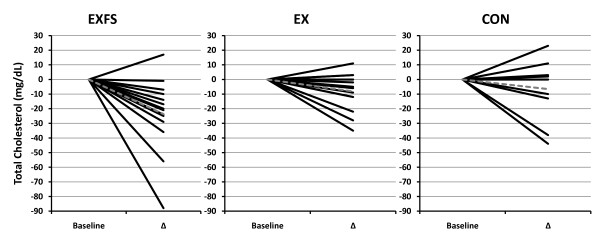
**Change in Total Cholesterol by Subject**. Individual responses for changes in Total Cholesterol (TC) from Baseline to Post (Δ). Exercise + FS (EXFS; left); Exercise-alone (EX; center); Control (CON; right). Dashed line represents the mean. TC was significantly reduced over time (*p *≤ 0.017) in EXFS.

**Figure 9:**
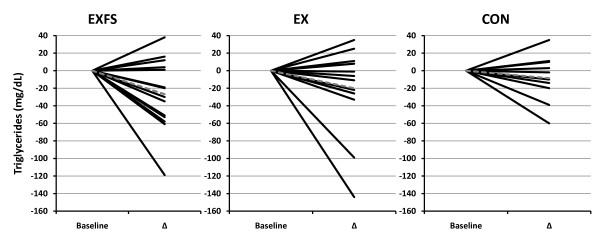
**Change in Triglycerides by Subject**. Individual responses for changes in Triglycerides (TRI) from Baseline to Post (Δ). Exercise + FS (EXFS; left); Exercise-alone (EX; center); Control (CON; right). Dashed line represents the mean. TRI did not change significantly across treatments (*p *≥ 0.05).

Fasting lipid concentrations are presented in Table 7, and individual responses for total cholesterol (TC) and triglycerides (TRI) in Figures 8 and 9. TC and low-density lipoproteins (LDL) decreased significantly in EXFS (-12.0% and -13.3%, respectively; *p *≤ 0.017), whereas no significant changes in TC and LDL were observed in EX or CON. The TC-to-high-density lipoprotein ratio (TC:HDL) decreased significantly in both EX and EXFS (-8.7% and -9.5%, respectively; *p *≤ 0.017), with no changes in CON.

## Discussion

As hypothesized, consumption of the food supplement (FS) significantly increased protein (PRO) and fiber intake in EXFS; PRO rising from 15.9% (86.3 ± 8.5 g/d; ~1.02 g/kg/d) of total energy at baseline (PRE) to 28.3% (131.3 ± 4.0 g/d; ~1.58 g/kg/d) during weeks 3–10. Simultaneously, subjects in EXFS realized a significant decrease in carbohydrate (CHO), fat (FAT) and total energy. Specifically, CHO decreased from 48.3% (261.5 ± 20.9 g/d) to 41.1% (190.3 ± 10.5 g/d) of total energy; FAT, from 35.8% to 30.6%; and total energy, from 2166 (± 160.0) to 1854 (± 98) kcals/d [Table 3]. The reduction in total energy intake may be explained by the satiating effect of PRO and/or fiber [18,19]; though it is our conclusion the modest increase (+4 g/d) in dietary fiber, albeit statistically significant, was not a major contributing factor. Instead, the observed non-significant increase in PRO, during weeks 1–2, and the significant decrease in CHO, FAT and energy intake over that same time period in EXFS would seem to support the separate hypotheses that CHO or FAT reduction spontaneously reduce energy consumption [20-22]. Such a conclusion runs contrary to findings by Weigle et al. [23] that increasing PRO-alone elicits a spontaneous reduction in *ad libitum *energy intake, or that only PRO intake, within EXFS, was significantly different from both control (CON) and EX during weeks 3–10. Instead, it is proposed that both an increase in dietary PRO and a reduction in CHO are equally necessary [24]; however, in accordance with our study design, we hypothesize that the decrease in CHO was predicated by the initial increase in PRO. Other possible explanations for the reduced energy intake are 1) the high viscosity of the nutritional shake [25], 2) added multivitamin and mineral supplementation [26], 3) reduced portion sizes [27], and/or 4) limiting variety and adding structure to the diet [28].

It has been well documented that, in the absence of dieting, exercise elicits only minor effects on total body mass (BM) despite significant improvements in cardiovascular fitness and strength [29,30]. King et al. ([31] propose that "inter-individual variability," or behavioral and metabolic compensatory events in response to exercise-induced increases in energy expenditure, may largely explain non-significant changes in BM from exercise-only interventions. In agreement, we found little individual variability in BM (92.9% of EXFS subjects' ΔBM occurred within +1.01 and -3.85 kg; 92.9% of EX subjects' ΔBM occurred within +2.39 and -2.17 kg). Individual responses for *ad libitum *energy intake (Figure 2) did, however, provide evidence of what appears to be an apparent trend toward compensatory increase in energy intake in 57.1% (n = 8) of subjects in EX but only14.3% (n = 2) of subjects in EXFS. Consequently, average energy intake was significantly reduced within EXFS (Table 3); supportive of the FS provoking a satiating effect. Additionally, Lofgren et al. [32] state that even modest changes in BM (<5%), in response to reduced energy and CHO intake and increased physical activity, improves cardiovascular health as assessed by low-density lipoprotein (LDL) cholesterol. In agreement, reductions in LDL only reached significance in EXFS (ΔBM = -2.13%; *p *> 0.05); the only group within the current study that realized a significant reduction in energy and CHO intake (Tables 3 and 7).

Expectedly, significant improvements in strength, cardiovascular fitness and blood lipids were observed in both EX and EXFS (Tables 5, 6, 7). Of special note, however, the change in time-to-exhaustion (TTE), in EXFS, was significantly greater than both CON and EX (EXFS = +118.7 sec > EX = +66.8 sec > CON = +9.1 sec; Table 6), with all subjects in EXFS resulting in a minimum improvement in fatigue threshold of +45 sec (Figure 7). Speculatively, the 100% improvement rate in TTE, within EXFS, may be attributable to the rise in muscle mass (MM; +2.3%) and greater reduction in fat mass (FM) [33], stable blood glucose, hormonal or other physiological adaptation [24], specific macro- and/or micro-ingredients of the food supplement or improved recovery nutrition between exercise bouts [34], improved hydration due to twice daily liquid supplementation, or possibly that the EXFS group was not blinded to the intervention. Further research controlling for such variables is warranted.

Interestingly, whereas neither EX or EXFS realized a significant reduction in plasma triglycerides (-16.1% and -19.4%, respectively), only EXFS experienced significant reductions in total cholesterol (-23.6 mg/dL) and LDL (-15.9 mg/dL) (Table 7 and Figures 8 and 9). The non-significant change in triglycerides, within EXFS, is noteworthy because the %Δ (-19.4%) is, in fact, consistent with findings involving low-carbohydrate and/or energy-restricted diets [6,7,32]. One explanation for the non-significant change may simply be sample size-dependent, whereas it is also plausible that EXFS achieved neither a great enough absolute reduction in CHO and/or energy to elicit such a response [35]. Layman and Walker [36], on the other hand, posit that both CHO must be below 150 g/d and PRO greater than 1.5 g/kg/d to elicit effective treatment against obesity and metabolic syndrome; only the latter was, in fact, achieved in EXFS (CHO = 190.3 ± 10.5 g/d; PRO ≈ 1.58 g/kg/d).

According to a recent meta-regression by Krieger et al. [4], the reduction in CHO to <41.4% of total energy and increase in PRO to >1.05 g/kg/d, observed within EXFS, can account for the 1.6 kg, 1.3%, 1.5 kg and 0.3 kg greater improvements in FM, percent body fat, BM and MM respectively, compared to EX [Note: The regression analysis by Krieger et al. stated an additional 0.60 kg of fat-free mass was associated with PRO intakes of >1.05 g/kg/d.]. Of particular value is that supplementation with FS reduced the variability in FM and MM responses to exercise, such that 100% of subjects in EXFS realized a significant improvement; a finding that would seem to support a metabolic advantage of low-carbohydrate/high-protein diet modification [37]. However, changes in CHO and PRO alone cannot, in the current study, be viewed in lieu of modifications in dietary FAT. According to a prediction equation developed by Astrup et al. [22], 1.17 kg of the 1.8 kg of FM lost by EXFS can be accounted for by the 26.71% reduction in dietary FAT. Thus, it seems prudent that future research incorporate isocaloric manipulations of varying macronutrient contributions such that contributing factors and covariates become more evident.

If, instead, we assume 0.45 kg of FM is equivalent to an ~3500 kcal deficit, the -2.7 kg change in FM, within EXFS, could almost completely be accounted for by the -312 kcal/d (-14.4%) reduction in energy intake:

$$\frac{3500kcals}{0.45kgFM}\times \frac{-2.7kgFM}{70days}=-300kcals/d$$

Such assumptions, simplified to "calories in versus calories out," would however fail to recognize the increased energy demands requisite for the +0.6 kg of MM observed within EXFS. Instead, a cumulative metabolic advantage, as postulated by Fine and Feinman [37] and reported by Scott and Devore [38], combined with the anabolic response to increased amino acid availability [24,39], and potentially sustained thyroid hormone levels and reduced insulin response [6] are more probable mechanisms to explain the significant mean changes in EXFS body composition measures. Layman et al. [39], for example, suggests that a hypocaloric diet with carbohydrate-to-protein ratio (CHO:PRO, in g/d) of 1.5:1.0 or less would be more effective in altering body composition than the 3.5:1.0 ratio currently recommended [39]. Consequently, these authors [39] reported decreases in FM (-22%) and no loss in lean body mass after 16 weeks of an energy-restricted diet composed of a CHO:PRO ratio of ~1.5:1.0, during an exercise program similar to that of the present study. Meckling and Sherfey [40] postulated similar conclusions in response to energy restriction and a 1:1 versus a 3:1 CHO:PRO ratio, with or without exercise, in overweight and obese women. It was reported that both a 1.5:1.0 diet-only and 0.96:1.00 diet + exercise treatment was more effective than traditional high carbohydrate, energy restriction-alone or with exercise. Our findings support these hypotheses and suggest that addition of the FS to the EXFS group's *ad libitum *diet lowered the CHO:PRO ratio from 3.03:1.00 to 1.46:1.00 for the 10-week training period and, as described by Wood et al. [21], spontaneously reduced *ad libitum *energy intake via an as of yet fully understood behavioral effect. This change may have accounted for the greater improvements and reduced variability of individual responses for FM and MM in EXFS, when compared to the 3.40:1.00 CHO:PRO ratio of EX. Another plausible explanation, though not directly assessed in the current study, may be that subjects in EXFS consumed one of the FS shakes as a breakfast meal; the addition of the second shake enabling for more frequent, protein-rich meals throughout the day. Such would be supportive of the hypothesis raised by Laymen [24]; that, consuming a minimum of 30 g of PRO for breakfast is "the most critical meal" for supporting an anabolic environment as is consuming PRO every 5–6 hours.

## Conclusion

In summary, in the absence of energy restriction or other dietary controls, provision of a commercially available high-protein/low-carbohydrate and fat, nutrient-dense food supplement (Full Strength^®^; EXFS), consumed daily, during a 10-week combined aerobic and resistance training intervention: 1) elicited a behavioral effect in previously sedentary, overweight adults such that subjects' macronutrient profiles were significantly modified (protein increased; carbohydrate and fat decreased) and total energy intake decreased spontaneously; 2) physiological adaptations to exercise were improved; and lastly, 3) though the significant mean differences between the exercise-only and EXFS groups may not be impressive in the absolute, consumption of the food supplement reduced the variability of individual responses for fat mass, muscle mass and time-to-exhaustion – all three variables improving in 100% of subjects in EXFS. This is of particular interest because virtually all of the available information pertaining to the use of food supplement interventions in overweight and obese populations has involved both an energy-restricted diet and the methodological replacement of at least one traditional meal per day [10]. It is recommended that future research explore the use of the food supplement under various isocaloric and controlled macronutrient ratios, as well as assess the potential impact of the added micronutrients and other ingredients, and whether or not meal timing and frequency are covariates to the improvements noted in body composition, fitness and cardiovascular health.

## Competing interests

The authors declare that they have no competing interests.

## Authors' contributions

CML: obtained funding, study design, intervention protocols, analysis and interpretation of data, and preparation of manuscript. JRM: study design, intervention protocols, acquisition of data, analysis and interpretation of data, and preparation of manuscript. SET: study design, intervention protocols, acquisition of data, analysis and interpretation of data, and preparation of manuscript. AAW: study design, intervention protocols, acquisition of data, analysis and interpretation of data, and preparation of manuscript. AES: study design, intervention protocols, acquisition of data, analysis and interpretation of data, and preparation of manuscript. VJD: intervention protocols, acquisition of data, analysis and interpretation of data, and preparation of manuscript. JTC: study design, intervention protocols, acquisition of data, analysis and interpretation of data, and preparation of manuscript. JRS: principal researcher, obtained funding, study design, intervention protocols, acquisition of data, analysis and interpretation of data, and preparation of manuscript. All authors read and approved the final manuscript.
